# Analysis of Waste Material from Enzymatic Hydrolysis of Extruded Arabica Coffee Beans from a Bioeconomy Perspective

**DOI:** 10.1021/acsomega.5c07362

**Published:** 2025-11-11

**Authors:** Raquel C Ribeiro, Rita C. Alves, Liliana E Santo, Anabela S G Costa, Susana Machado, M. Beatriz P. P. Oliveira, Ricardo S S Teixeira, Claudia M. Rezende

**Affiliations:** † 28125 Universidade Federal do Rio de Janeiro, Instituto de Química. Rua Athos da Silveira Ramos, 149, 6° andar, 626A - Cidade Universitária, Rio de Janeiro 21941-909, Brasil; ‡ 131671 Universidade do Porto, Faculdade de Farmácia. Rua Jorge Viterbo Ferreira, 228, Porto 4050-313, Portugal; § Universidade Federal do Rio de Janeiro, Instituto de Química. Rua Moniz de Aragão, 1057 − Cidade Universitária, Rio de Janeiro 21941-914, Brasil

## Abstract

The growing demand for sustainable and innovative approaches to agro-industrial waste management has drawn particular attention to the coffee industry, one of Brazil’s most prominent agricultural sectors. This study applied the biorefinery concept to defective coffee beans, unsuitable for direct consumption, using healthy coffee beans as a reference. Processes such as extrusion, oil extraction, and enzymatic hydrolysis were performed to recover carbohydrates. After hydrolysis, several analyses were performed, revealing significant differences in the nutritional parameters and bioactive compounds. These analyses were very important because they allowed the correct designation of the use of these residues. Approximately 60% of mannose and fructose were recovered in both types of beans. The hydrolyzed residues from defective and healthy beans maintained high protein (∼15%) and fiber content. The defective beans also showed significantly higher levels of free amino acids, flavonoids, and antioxidant capacity, indicating the preservation of bioactive compounds. The results highlight the feasibility of using these residues in the development of functional products or nutritional supplements.

## Graphical abstract

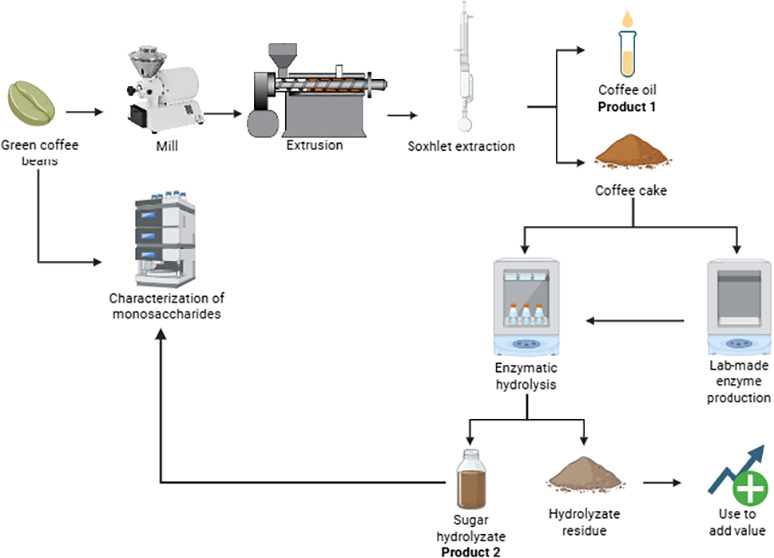

## Introduction

Sustainability and the pursuit of innovative solutions for the use of agro-industrial waste have become central themes in fostering a more circular and integrated economy.^1^ Inside the concept of bioeconomy the biorefinery stands out, which proposes the maximum use of byproducts from manufacturing processes, minimizing waste and adding economic and environmental value.^2^ The use of byproducts in the food industry enables a valuable opportunity to mitigate environmental impacts while simultaneously creating added economic value.^3,4^ A promising example is the use of coffee bean waste, like the coffee husks and defective beans, which traditionally have lower commercial value.^5^

Coffee, one of the world’s most widely consumed beverages, generates a considerable amount of waste in its various processing stages. The coffee industry generates around 40 million tons of biological waste per year,^6^ which includes spent coffee grounds, coffee husks, defective beans, and others.^7,8^ Defective beans represent around 20% of global coffee production, which directly impacts countries like Brazil, the world’s leading producer of the raw coffee beans.^9^

Traditionally, defective coffee beans are blended with higher-quality ones for distribution within the domestic market in Brazil, as they fail to meet the quality standards required for international trade.^10,11^ Although they negatively affect beverages, these beans still contain bioactive compounds and nutrients that can be applied in different industrial areas,^12–15^ and this approach aligned with the principles of the circular economy.

Several pretreatment strategies have been developed to enhance biomass in valuable products.^16^ Extrusion is distinguished as a physical pretreatment that modifies the structural properties of biomass without altering its chemical composition.^17^ This technique presents several advantages, such as particle size reduction, increased surface area of contact, and the absence of byproduct or toxic residue formation. Moreover, when combined with subsequent enzymatic hydrolysis, extrusion can enhance the release of target compounds, contributing to a more efficient biomass conversion.^18,19^ Enzymatic hydrolysis is an approach that breaks down complex molecules, widely used to recover different types of sugar from plant biomass.^17,20,21^ Monosaccharides obtained from the enzymatic hydrolysis of complex carbohydrates have great potential that can be used as raw materials in various industries, including the food, pharmaceutical, and chemical industries.^22^

The addition of the biorefinery concept to the coffee chain has multiple benefits. As well as reducing waste disposal, this approach promotes the economic valorization of neglected byproducts. In a preliminarily extrusion optimization, it was investigated coffee oil extraction as shown by Ribeiro et al.,^12^ resulting in patent application submitted to Instituto Nacional da Propriedade Industrial - INPI (BR10202302074). In this new article, the research was expanded to a circular economy concept, using the waste generated by the extrusion process followed by degreasing to recover monosaccharides through enzymatic hydrolysis. In addition, the final waste from this process is labeled in terms of nutritional parameters and bioactive compounds. Therefore, in addition to these innovative results, we also propose the creation of a biorefinery, contributing not only to the sustainability of the coffee production chain but also to the development of new products and economic opportunities, paving the way for future applications and technological advances.

## Experimental Section

The experimental procedure was carried out using the methods described below, based on preview results of Ribeiro et al.^12^

### Reagents

Hexane was obtained from Neon (São Paulo, BR). Sodium citrate was from Exodo Científica (São Paulo, BR). Sodium azide was purchased from Vetec (Rio de Janeiro, BR). Chlorogenic acid, (±)-catechin hydrate, iron­(II) sulfate heptahydrate, sodium nitrite, aluminum chloride hexahydrate, 2,4,6-tris­(2-pyridyl)-s-triazine (TPTZ), 2,2-diphenyl-1-picrylhydrazyl (DPPH), (±)-6-hydroxy-2,5,7,8-tetramethylchromane-2-carboxylic acid (Trolox), and iron­(III) chloride hexahydrate, d-glucose, d-galactose, d-mannose, l-arabinose, d-xylose, and d-fructose, total dietary fiber assay kit, Celite, and amino acid standards (with purity grades ranging from 98.0% to ≥ 99.5%) were all obtained from Sigma–Aldrich (St. Louis). Anhydrous sodium carbonate, sodium hydroxide, absolute ethanol, Kjeldhal tablets (without addition of Se and Hg), and anhydrous disodium hydrogen phosphate, and disodium tetraborate decahydrate were from Merck (Darmstadt, Germany). Petroleum ether, sodium dihydrogen phosphate, sodium hydroxide, and sulfuric acid were from Carlo Erba Reagents (Val de Reuil, France). Boric acid was from Chem-Lab (Zedelgem, Belgium), and sulfuric acid was from Honeywell Fluka (Düsseldorf, Germany). Borate buffer (0.4 N, pH 10.2), o-phthalaldehyde/3-mercaptopropionic acid (OPA/3-MPA), and 9-fluorenylmethylchloroformate (FMOC) were all from Agilent Technologies (California). Sodium azide, methanol, and acetonitrile were from Honeywell Riedel-de Han (Seetze, Germany). Potassium hydroxide and hydrochloric acid (≥37%) were obtained from Fisher Chemical (Belgium). Water was purified in a Milli-Q system (Millipore, Bedford, Massachusetts). All of the reagents used were analytical grade.

### Coffee Beans

The raw arabica coffee beans (*Coffea arabica* L.) of the Catua amarelo cultivar were harvested from April to August, 2018, in São Francisco farm, located in São José do Vale do Rio Preto, Rio de Janeiro, Brazil (22°11′35.2″S, 42°59′8.6″W). The beans were collected from 10 different bags of 60 kg, sampled and sent for separation of the defective beans, allowing a highly diversity in sampling of the coffee from this production site.

According to the Official Classification Table,^23^ 300g of coffee beans was separated according to their defects. The material was then grouped into healthy (no defects) and defective (black, green, burnt, broken, badly grained, shells, and shell kernels).

The coffee beans were grinded in a knife mill (Retsch SM 300, Germany) (18 mesh, 1 mm diameter) as described by^24^ for the chemical and nutritional analyses.

### Characterization of Coffee Beans

#### Moisture, Ash, and Extractive Content

Moisture was determined in a Heratherm OGS oven (Thermo Scientific, Germany) at 105 °C until constant weight.^24^ The ash content was measured using a muffle furnace (Lindberg Blue M, Thermo Scientific, Germany) at 545 °C, and the extractive content was measured by extracting the compounds with hexane, water, and ethanol using Soxhlet.^25^

#### Monosaccharide Content by Acid Hydrolysis

Free carbohydrates in the bean were determined by acid hydrolysis.^26^ A 300 mg sample of ground coffee was mixed with 3 mL of 72% sulfuric acid and placed in a water bath at 30 °C for 60 min. The samples were then diluted with 84 mL of distilled water and autoclaved at 121 °C for 1 h.

After the acid hydrolysis, the samples were vacuum filtered and an aliquot of the filtered material was neutralized with calcium carbonate, reaching a pH between 5 and 6. The samples were centrifuged at 5000 rpm for 10 min, and the supernatant was analyzed by HPLC-RI, with 10 μL of sample injected into a Dionex Ultimate 3000 system (Thermo Scientific, Germany) equipped with a refractive index detector RefractoMax 521 (Thermo Scientific, Germany), using an Aminex HPX-87P column (300 mm × 7.8 mm, 9 μm, Bio-Rad). The mobile phase was 100% Milli-Q water at a flow rate of 0.6 mL·min^–1^. The total run time was 25 min. Quantification was carried out using an external analytical curve with a mixture of sugar standards as d-cellobiose (1.9 mg·mL^–1^), d-glucose (6.2 mg·mL^–1^), d-xylose (2.98 mg·mL^–1^), d-galactose (2.12 mg·mL^–1^), l-arabinose (2.95 mg·mL^–1^), and d-mannose (4.91 mg·mL^–1^) (>98%), with 2, 4, 8, and 16-fold dilutions. Mannose and fructose content were described together due to limitations of the analytical procedure.

Carbohydrate concentration (mg·mL^–1^) is represented in eq 1, where “Chplc” represents the sugar concentration determined by HPLC, “D” represents the sample dilution, and “R” represents the average recovery of the specific sugar standard. Carbohydrate concentration (g·100 g^–1^ of bean) considering the extractive content is represented in eq 2, where “Cx1” represents the concentration of sugar (mg·mL^–1^) in the hydrolyzed and “Ex” represents the percentage of extractives.

Determination of carbohydrates in mg·mL^–1^.

1 $${Cx}_{1}\left({mg\cdot mL}^{-1}\right)=\frac{Chplc\cdot D}{\frac{R}{100}}$$

Determination of carbohydrates in g·100 g^–1^ of bean.

2 $${Cx}_{2}\left(\%\right)={Cx}_{1}\cdot \frac{100-Ex}{100}$$

### Processes Applied to Coffee Beans

#### Extrusion

Coffee beans were extruded in a twin-screw extruder RheoDrive 7 OS e Rheomex PTW 16/25 OS (HAAKE PolyLab OS, Thermo Scientific, Germany) with a temperature of 68 °C and a screw rotation speed of 60 rpm, for 3 cycles.^12^

#### Soxhlet

After extrusion, coffee oil was removed from the biomass as a product within the biorefinery concept. The lipids were extracted by Soxhlet using hexane for 4 h and the solvent removed in a rotary evaporator.^12^

#### Enzymatic Hydrolysis

The extruded and defatted samples were subjected to enzymatic hydrolysis using 12% dry biomass. The experiments were conducted at 50 °C and pH 4.8 (100 mM sodium citrate buffer) for 72 h. In addition to the temperature and pH conditions, the Shaker-type incubator (Innova 44R, New Brunswick) was set to 200 rpm, and in order to inhibit the growth of microorganisms and avoid contamination, a small amount of sodium azide was added to the media. A proportion of 15 U·mL^–1^ of an enzyme extract obtained by cultivating *Aspergillus niger* 1234 using coffee cake as a substrate was used in the hydrolysis process 11.

After the hydrolysate, which contained carbohydrates (another product in the biorefinery concept), was removed, the solid biomass was studied to determine its physicochemical composition.

### Chemical Characterizatoin of the Enzymatic Hydrolysate

The carbohydrates obtained by enzymatic hydrolysis were determined by putting 2 mL sample of the hydrolysate in a water bath at 100 °C for 5 min in order to stop the reaction. The material was then centrifuged at 8000 rpm for 10 min and analyzed by HPLC under the same conditions as described previously.

### Analysis of the Residual Coproducts from Hydrolysis

#### Nutritional Analysis

The moisture content was determined on an infrared balance (DBS-KERN & SOHN, Balingen, Germany) at 105 °C. The remaining nutritional analyses were performed using AOAC standardized methods:^28^ the ash content was determined by incineration (Thermolyne Muffle Furnace, model 48000, Suwanee, GA) at 500 °C; the total fat content was determined by the Soxhlet extraction method, using petroleum ether for 8 h. Crude protein was determined using the Kjeldahl method and the nitrogen (N) conversion factor of 5.24.^27^ The total and insoluble fiber contents were analyzed by enzymatic-gravimetric methods. Soluble fiber and available carbohydrates were calculated by difference.^29^

#### Nitrogen Content

Protein nitrogen content was determined by mixing 200 mg of sample with 20 mL of deionized water and, after homogenization, left to stand for 30 min. Then, 4 mL of trichloroacetic acid (TCA) solution (15%) was added, and the stirring and resting process was repeated. The mixture was then filtered through low nitrogen filter paper (GLP, Portugal) and the residue was washed with 15% TCA.^30^ The fraction retained on the filter (protein fraction) was then subjected to acid digestion^31^ according to the Kjeldahl method. Nonprotein N was determined by the difference between the total N value and the protein N value.

#### Caffeine and Chlorogenic Acids

Caffeine content of the extracts was analyzed using an HPLC-DAD system (Jasco, Tokyo, Japan), with a Zorbax-SB-C18 column (5 μm, 250 mm × 4.6; Agilent Technologies) at 28 °C. The gradient solvent system with a flow rate of 1.1 mL·min^–1^ consisted of A (CH_3_COOH/H_2_O 0,5:99,5) and B (MeOH), according to 5% B at 0 min; 25% B at 40 min; 45% B at 55 min; 60% B at 60 min; and 5% B at 65 min. The injected volume was 20 μL, monitored at 274 nm for caffeine and 320 nm for chlorogenic acids (3-, 4-, and 5-caffeoylquinic acids). Calibration curves were prepared with standards (caffeine: 1.5–800 mg·L^–1^; chlorogenic acids: 1.0–259 mg·L^–1^). Compounds identification was performed by comparing retention times and coelution with authentic standards, and by UV absorption spectral analysis.^27^

#### Amino Acids

Alkaline hydrolysis was carried out to determine the tryptophan content as described by ref.^30^ Briefly, 150 g of sample was mixed with 3 mL of 4 M KOH. The mixture was heated in a thermal block (SBH130*D*/3, Stuart, Stafford, UK) at 110 °C for 6 h. After centrifugation for 10 min at 5000 rpm (Labofuge Ae centrifuge, Heraeus Sepatech, Germany), 50 μL of the supernatant was collected and mixed with 940 μL of 0.1 M HCl and 10 μL of internal standard (norvaline, 2 mg·mL^–1^). The mixture was centrifuged again, and 950 μL of the supernatant was taken to automatic derivatization (AS-4150 autosampler, Jasco, Japan).

Acid hydrolysis was carried out to determine the remaining amino acids content.^30^ The sample (150 mg) was mixed with 3 mL of 6 M HCl and heated in a heat block (SBH130*D*/3, Stuart, Stafford, UK) at 110 °C for 24 h. After centrifugation for 10 min at 3500 rpm (Labofuge Ae centrifuge, Heraeus Sepatech, Germany), 50 μL of the supernatant was collected and mixed with 940 μL of borate buffer (pH 10.2) and 10 μL of internal standard (norvaline, 2 mg·mL^–1^). The mixture was centrifuged again, and 950 μL of the supernatant was automatically derivatized (AS-4150 autosampler, Jasco, Japan).

For the extraction of free amino acids, 0.5 g of the sample was mixed with 10 mL of deionized water and subjected to magnetic stirring for 30 min at 40 °C. The mixture was then centrifuged at 3500 rpm for 10 min using a Labofuge Ae centrifuge (Heraeus Sepatech, Germany). The resulting residue was subsequently extracted again with an additional 5 mL of deionized water under the same conditions for 15 min. Following a second centrifugation stage, the supernatants from both extractions were combined for chromatographic analysis. An aliquot of 1.5 mL of the pooled supernatants was centrifuged at 13000 rpm for 10 min (Heraeus Fresco 17 centrifuge, Thermo Fisher Scientific, Germany). Then, 990 μL of the resulting supernatant was mixed with 10 μL of internal standard (norvaline, 2 mg·mL^–1^) and loaded into an AS-4150 autosampler (Jasco, Japan) for automatic derivatization.^30^

Free and total amino acid profiles were analyzed in an integrated system from Jasco (Tokyo, Japan), with a ZORBAX Eclipse Plus C18 column (4.6 × 250 mm, 5 μm, Agilent Technologies) at 40 °C. The gradient solvent system with a flow rate of 1.5 mL·min^–1^ consisted of A (phosphate/borate buffer - Na_2_HPO_4_ 10 mM: Na_2_B_2_O_7_ 10 mM (pH = 8.2): NaN_3_ 5 mM) and B (MeOH:ACN:H_2_O – 45:45:10), according to 2% B at 0.85 min; 57% B at 33.4 min; 85% B at 39.3 min; 2% B at 40 min. Fluorescence signals were monitored in excitation at 340 nm and emission at 450 nm, from 0 to 26.2 min for o-phthaldehyde (OPA) derivatives, and in excitation at 266 nm and emission at 305 nm, from 26.2 to 40 min for fluorenylmethylchloroformate (FMOC) derivatives.^30^

#### Antioxidants

250 mg of ground sample (Grindomix GM 200, Retsch, Haan, Germany) was mixed for 1 h with 20 mL of ethanol/water solution (1:1) at 2000 rpm (Multi Reax, Heidolph Instruments, Schwabach, Germany). After centrifugation at 5000 rpm for 5 min (Labofuge Ae centrifuge, Heraeus Sepatech, Germany), the supernatant was collected and the residue was extracted again under the same conditions. After the supernatants were combined, an aliquot was centrifuged at 13000 rpm for 10 min (Heraeus Fresco 17 centrifuge, Thermo Fisher Scientific, Germany) and further analyzed as described below.

The total phenolic content was estimated by spectrophotometry using a Biotek Synergy HT microplate reader (BioTeK Instruments Inc., Winooski, Vermont),^27^ in which a 30 μL extract aliquot (1:10) was mixed with 150 μL of Folin-Ciocalteu reagent (1:10) and 120 μL of a Na_2_CO_3_ solution (7.5% w/v). The mixture was incubated at 45 °C for 15 min and then left at room temperature for 30 min. The absorbance was measured at 765 nm. The total phenolic content was calculated from a calibration curve prepared with chlorogenic acid (5–160 mg·L^–1^) and expressed as mg of chlorogenic acid equivalents (CAE) g^–1^ of sample.

Total flavonoids content was determined by spectrophotometry,^32^ in which 1 mL of extract was mixed with 4 mL of distilled water and 300 μL of 25% NaNO_2_. A 300 μL amount of 10% AlCl_3_ was added to the mixture after 5 min at room temperature, and after 1 more minute, 2 mL of 1 M NaOH and 2.4 mL of ultrapure water were added. The absorbance was measured at 510 nm (Synergy HT, BioTeK Instruments. Inc., Winooski, Vermont) and the total flavonoid content was estimated using an catechin calibration curve (5–100 mg·L^–1^) and expressed as mg of catechin equivalents (CE) L^–1^ of extract.

The FRAP solution was prepared with 0.3 M acetate buffer, 10 mM 2,4,6-tris­(2-pyridyl)-(S)-triazine (TPTZ), and 20 mM FeCl_2_. For the assay, 30 μL of diluted extract (1:50) was mixed with 270 μL of FRAP solution. After homogenization, the mixture was kept for 30 min at 37 °C and the absorbance was measured at 595 nm (Synergy HT microplate reader, BioTeK Instruments. Inc., Winooski, Vermont). A calibration curve was prepared with ferrous sulfate (50–600 μmol L^–1^) and the ferric reducing antioxidant power was expressed as μmol of ferrous sulfate equivalents (FSE) g^–1^ of sample.^27^

The radical inhibition capacity of the extracts was analyzed according to,^27^ in which 30 μL of the diluted extract (1:10) were mixed with 270 μL of a freshly prepared DPPH (2,2-diphenyl-1-picrylhydrazyl radical) radical spot solution (6·10^–5^ mol L^–1^ in ethanol). The decrease in the DPPH radical spot was measured at equal time intervals of 10 up to 40 min, monitoring the decrease in absorbance at 525 min. A calibration curve was prepared with trolox (25–175 mg·L^–1^) and the DPPH radical scavenging activity was expressed as mg of trolox equivalents (TE) g^–1^ of sample.

#### Statistical Analysis

Data are reported as the mean ± standard deviation. Statistical treatment was performed using IBM SPSS Statistics 26 software (IBM Corp., Armonk, NY). Student’s test for independent samples was used to discriminate between any two groups under consideration.

## Results and Discussion

### Characterization of Coffee Beans

#### Moisture, Ash, and Extractive Content

The moisture content for healthy and defective beans was 6.94 ± 0.53% and 8.95 ± 0.04%, respectively, and the ash content was 4.03 ± 0.03% and 4.67 ± 0.11%, respectively, in agreement with Ribeiro et al.^12^ Both values are close to those reported in the literature, where the moisture content ranges from 7.9 to 13% and the ash content ranges from 1.7 to 6%.^10,33–36^

The extractive content was 52.55 ± 0.48% for healthy beans and 58.22 ± 6.90% for defective beans. Extractive content refers to compounds that are not associated with the plant cell wall and have a low or a medium molar mass. These chemical compounds can be extracted using water or organic solvents. Salts, organic minerals, sugars and polysaccharides are generally soluble in water, while substances such as organic acids, fatty esters, long-chain alcohols, steroids, phenolic compounds and glycosides dissolve in organic solvents.^37^ In this study, the extractive content was investigated in order to correct the calculation of the monosaccharides present in coffee beans.

#### Monosaccharides Obtained through Acid Hydrolysis

Poisson et al.^38^ reported that the mannose content of raw Arabica coffee beans ranges from 10 to 20%. Oestreich-Janzen (2010)^39^ showed that monosaccharides in raw Arabica coffee were 19.85% for mannose, 10.37% for galactose, 9.35% for glucose, 3.92% for arabinose and 0.22% for xylose. Both coffee beans analyzed in this study, whose values are presented in Table S1, had a mannose content similar to the previous works: 19.69% for healthy and 18.50% for defective. However, in relation to the other monosaccharides, it was observed in Table S1 that the coffee under study had a higher content of galactose and reduced levels of glucose and arabinose, while xylose was not detected. According to Redgwell and Fischer^40^ and Portillo and Arévalo,^41^ discrepancies in monosaccharide values can be attributed to different techniques used for extracting and determining (identifying and quantifying) the analytes.

### Oil Obtained by Extrusion Followed by Soxhlet

As the first product in the biorefinery field, oil production was investigated. As previously discussed in Ribeiro et al.,^12^ an increase in oil content was observed when the beans were pretreated by extrusion followed by Soxhlet extraction in comparison with not pretreated (only Soxhlet). In healthy coffee beans, the percentage of lipids increased from 9.05 to 16.42%, while in defective beans, this content increased from 9.47 to 15.29%, when extrusion was carried out with optimized parameters of 68 °C and 60 rpm. This could be explained by the rupture of the cell wall caused by the shearing of the material in the extruder and, consequently, the rupture of the lipid pockets as well as the reduction in the granulometry of the material and the increase in the contact surface, allowing greater permeability of the solvent in the extraction process. These results also led to the patent application submitted to the Instituto Nacional da Propriedade Industrial (INPI) on October 6, 2023 (BR10202302074).

After the oil is removed, the remaining solid residue (coffee cake) can be used to obtain fermentable sugars like glucose and mannose, through enzymatic hydrolysis.

### Chemical Characterization of the Enzymatic Hydrolysate

Pre-established conditions of 50 °C, 200 rpm, and pH 4.8 for enzymatic hydrolysis were chosen based on studies described in the literature, in which a temperature of 50 °C and pH 4.8 are often reported as suitable for this type of process, while stirring at 200 rpm favors enzyme–substrate contact, increasing enzymatic activity.^42–46^

During enzymatic hydrolysis, the polysaccharides are broken into monosaccharides, mainly mannose, glucose, and galactose, and become accessible for use in other biotechnological processes. For extruded and defatted healthy and defective coffee beans after 72 h of enzymatic hydrolysis, no significant difference (*p* > 0.05) was observed in the results, described in Table S2.

Enzymatic hydrolysis was able to recover 59 and 63% of the mannose+fructose content in the healthy and defective beans, respectively. Although the extrusion pretreatment was optimized for lipid recovery rather than sugars, this result indicates its effectiveness and the high capacity of the enzyme to convert the mannan polysaccharides present in the coffee bean. This was expected, given the high activity of the mannanases mentioned above.

The high recovery of glucose also demonstrates the activity of other hydrolases present along with the mannanases. The enzymes were not effective in recovering galactose and arabinose, so it can be concluded that type II arabinogalactan present in the beans was not hydrolyzed. Regarding the total recovery of sugars, the efficiency of the enzymatic process was approximately 42% for both beans, suggesting that the enzyme can be added to a specific commercial enzyme for converting galactose and arabinose. Furthermore, it is suggested that an in-depth study be carried out to optimize the extrusion process for monosaccharide recovery, since our process was optimized to recover lipids.

The solid residue resulting from the hydrolysis process, which consisted of 54.27 ± 3.02% dry mass, was subjected to nutritional analysis.

### Analysis of the Residual Coproducts after Hydrolysis

#### Chemical Composition

The compositional analysis of the residual coproducts derived from healthy and defective coffee beans revealed significant differences in several nutritional parameters and bioactive compounds, present in Table S3. Ash, lipid, and fiber contents were significantly higher (*p* < 0.05) in the sample obtained from healthy beans when compared to those from defective ones, while the remaining carbohydrates content, estimated by difference, showed an opposite trend, although the level of intensity from the differences is relatively small.

For the crude protein content, no significant difference (*p* > 0.05) was observed between the samples, with values around 15% for both (Table S3), in agreement with Zhu et al.^47^ for healthy Arabica coffee beans from different origins (13.60–15.27 g·100 g^–1^) and by Machado et al.^27^ for defective coffee beans (13.28 g·100 g^–1^). This suggests that the processes applied in this study (extrusion, Soxhlet, and enzymatic hydrolysis) did not extract their protein content from the beans, and therefore, this material can be extracted as a new product for the biorefinery chain.

The fiber content was significantly higher (*p* < 0.05) in the coproduct derived from healthy coffee beans, around 56%, when compared to that from defective coffee beans, around 50% (Table S3), which suggests a modification in structural carbohydrates possibly due to prefermentation processes or microbial degradation in the defective samples. Despite the small difference, Penna^48^ described a total fiber content for healthy and defective Arabica coffee beans ranging from 49.88 to 62.69 g·100 g^–1^, and Machado et al.^27^ reported a value of 57.00 g·100 g^–1^ for defective coffee beans. The fiber contents found in the coproducts herein are consistent with those described for raw beans, indicating that although enzymatic hydrolysis was effective in recovering mannose, the beans still have a high carbohydrate content after this process. This result demonstrates low efficiency of the conversion of arabinogalactans which, according to Arya and Rao,^49^ represent a large part of the beans’s polysaccharides. Therefore, the protein and fiber fractions of hydrolyzed coproducts warrant further investigation to enhance their valorization. It is worth mentioning that the simultaneous presence of polysaccharides, proteins, and minerals turns coffee waste into products of high biotechnological value, which can be used as substrates in fermentation processes, for the cultivation of microorganisms and to produce compounds in the food and pharmaceutical sectors.^50,51^ According to Iriondo-DeHond et al.,^52^ fiber stands out as the main constituent in all coffee byproducts, notable for its lack of allergenic proteins and its ability to be easily extracted for use as a food ingredient. In addition, protein, as the second most prominent component in coffee byproducts, could also be isolated and used as a food ingredient.

The content of chlorogenic acid, described in Table S3, is significantly higher (*p* < 0.05) in samples derived from defective beans, while caffeine levels did not differ significantly (*p* > 0.05) between the two groups. In defective beans, Machado et al.^27^ found a caffeine content of 1.40 g·100 g^–1^, close to the results obtained in this study. Part of the caffeine is extracted along with the coffee oil during the Soxhlet process due to its interaction with the solvent, so it is acceptable for the residual coproduct to have a low concentration of this compound. Although the content was lower, it is still within the range described in the literature for coffee beans, between 0.6 to 1.8 g·100 g^–1^.^53–55^

Regarding caffeoylquinic acids, in healthy beans, the value found was 2.65 g·100 g^–1^ and in the defective beans, it was 3.59 g·100 g^–1^ (Table S3). Silva et al.^56^ found a content of 4.37 and 4.42 g·100 g^–1^ for chlorogenic acid in healthy and damaged beans, respectively; and Farah and Donangelo^57^ previously stated that in defective beans, the content of 3-CQA and 4-CQA increases in comparison to the levels of standard quality beans, coinciding with what was found in this study. Machado et al.^27^ found a content of 0.40 g·100 g^–1^ for 3-CQA, 0.68 g·100 g^–1^ for 4-CQA, and 3.79 g·100 g^–1^ for 5-CQA for chlorogenic acid in defective beans, values very close to those found in this study, except for the 5-CQA content, which was lower.

#### Amino Acids

The analysis of total and free amino acids revealed significant differences between the residual coproducts derived from healthy and defective coffee beans, described in Table S4. Regarding total amino acids, histidine, arginine, and tryptophan contents were significantly lower (*p* < 0.05) in defective samples, while an increase was observed for six amino acids (threonine, alanine, methionine, isoleucine, leucine, and proline). The total content of essential amino acids (∑EAA) also showed a nonsignificant increase in defective samples (63.14 mg·g^–1^) compared to healthy ones (58.50 mg·g^–1^).

In opposition, the free amino acids profile displayed more pronounced and significant alterations. Notably, several amino acids (such as glutamic acid, asparagine, and valine) were significantly elevated in defective samples (*p* < 0.05), suggesting protein degradation or metabolic imbalance in the defective beans. With a lesser or greater magnitude, this trend was observed across the majority of the free amino acids analyzed. In all amino acids, only four showed significantly higher contents (*p* < 0.05) in healthy grains (i.e., aspartic acid, histidine, arginine, proline) (Table S4). These overall results are consistent with reports in the literature, indicating that processing defects and cellular stress may lead to proteolysis and accumulation of certain free amino acids. Such findings are in accordance with those mentioned by Casas et al.^58^ that found an enriched profile in reducing sugars and free amino acids in defective coffee beans. It is important to highlight that the presence of key precursors such as asparagine and reducing sugars makes this composition particularly prone to acrylamide formation, especially if thermal processing is foreseen in future applications. Acrylamide is primarily formed through the Maillard reaction between free asparagine and carbonyl compounds at high temperatures;^59^ therefore, the significantly higher levels of free asparagine in the coproduct resulted defective beans may facilitate the potential formation of this undesirable compound, raising concerns from a food/feed safety perspective.

Given that the reference levels for acrylamide in roasted coffee are 400 μg·kg^–1^ and in instant coffee 850 μg·kg^–1^, with variations for other coffee-based products—defined by Commission Regulation (EU) 2017/2158 of November 20, 2017 ^60^—it is important to monitor the chemical composition of these byproducts, especially when their potential application involves thermal processing, such as in the use of roasted ingredients, flavoring agents, or functional food components.

Regarding the total concentration of free amino acids, the defective beans coproduct exhibited significantly higher levels (3664 μg·g^–1^) compared to their healthy counterpart (2232 μg·g^–1^). A similar trend was also observed when considering only essential amino acids (∑EAA: 1703.82 vs 938.70 μg·g^–1^, respectively), highlighting the potential of this defective material as a comparatively richer source of bioavailable amino acids. Despite their lower nutritional quality in terms of intact proteins, the higher presence of free amino acids could be advantageous for specific applications. In particular, nonthermal uses such as enzymatic hydrolysates, fermentation substrates, or functional ingredient formulations could benefit from this enhanced amino acid profile.

In a recent study, Dong et al.^61^ determined 13 amino acids in Arabica coffee beans, six of which were nonessential (Asn, Asp, Ser, Glu, Ala, Tyr) and seven essentials (Val, Ile, Leu, Phe, Lys, His, Arg). In the present study, more amino acids were analyzed, including four nonessential (Gln, Gly, Hyp, Pro) and three essentials (Met, Thr, Thp). The total content of free amino acids (TAA) determined for Arabica coffee beans ranged from 5419.8 to 9226.5 μg·g^–1^. The same was described by Hamzalıoğlu and Gökmen,^62^ who reported that the TAA content in Arabica coffee beans was 5.98 mg·g^–1^, which was higher than those found for the coproducts streaming from healthy and defective beans in this work. This suggests that free amino acids are partially extracted from the original matrices by the processes employed in this study.

As evidenced by several studies, glutamic acid is the major amino acids in coffee samples.^61,63,64^ The same was observed in this study. It was also highlighted a high concentration of leucine in the samples, which has already been described by Lago et al.,^65^ making these amino acids amenable to extraction in order to valorize the residue. For example, glutamic acid is the main component of the additive monosodium glutamate, which is widely used in the food industry as a flavor enhancer.^66^ In addition, some studies have shown the potential of leucine to stimulate muscle protein synthesis, and it is widely used in protein formulations, as well as to prevent sarcopenia in the elderly.^67^ Leucine also has powerful anabolic properties, acting as an insulin-secreting agent.^68^

#### Antioxidants

Phenolic compounds and alkaloids represent important bioactive compounds found in coffee beans. Their regular and appropriate consumption has been associated with health benefits, with phenolic compounds, in particular, being recognized as antioxidant agents, which may contribute to reducing the incidence of chronic diseases and, partially, cancer.^69^ In addition, the major coffee phenolics (chlorogenic acids) have been associated with antidiabetic effects.^70,71^

In this study, the total phenolic and total flavonoid contents were assessed as well as the antioxidant activity using both the DPPH inhibition and the ferric reducing antioxidant power (FRAP). These two methods were selected due to their complementarity nature regarding their mechanisms of action. The FRAP (Ferric Reducing Antioxidant Power) assay is based on the reduction of a ferric ion (Fe^3+^) complexed with 2,4,6-tripyridyl-(S)-triazine (TPTZ), resulting in the formation of a blue-colored ferrous complex that can be measured spectrophotometrically. This assay is particularly effective for assessing antioxidants with redox potentials below 0.7 V, which corresponds to the redox potential of the Fe^3+^-TPTZ complex. As such, it serves as a valuable *in vitro* tool for estimating the electron-donating capacity of compounds and their potential to support redox homeostasis in biological environments. Since the FRAP method relies solely on an electron transfer mechanism, it does not detect antioxidants that act exclusively via hydrogen atom transfer. On the other hand, the DPPH (2,2-diphenyl-1-picrylhydrazyl) radical scavenging assay can assess antioxidants capable of quenching free radicals through either electron donation or hydrogen atom transfer. This method evaluates antioxidant activity by monitoring the decrease in absorbance of the DPPH solution, which indicates the extent of radical neutralization.^72^

In this study, no significant difference (*p* > 0.05) was observed between the total phenolic content of the two samples, with mean values of 44 mg CAE·g^–1^ for the healthy bean residue and 41 mg CAE·g^–1^ for the defective one. However, the total flavonoid content was significantly higher (*p* < 0.05) in the defective bean residue (15.14 mg CE·g^–1^) compared to the healthy counterpart (9.60 mg CE·g^–1^), described in Table S5. In what concerns to the bioactive compounds, a wide range of values has been reported for phenolics and flavonoids in coffee beans. Phenolics have been reported to range between 1.47 and 190 mg GAE·g^–1^,^73–76^ while flavonoids show a range of 2.86 to 152.14 mg·g^–1^.^61,76,77^ Under the conditions evaluated, degradation of bioactive compounds is not expected to occur, since the processes applied (extrusion, Soxhlet extraction, and hydrolysis) were conducted at moderate temperatures (around 60 °C), which do not promote significant degradation of phenols or flavonoids. The absence of statistical differences in the total phenolic content between healthy and defective grains confirms that processing did not differentially affect these compounds. The higher flavonoid content observed in defective beans is, therefore, due to the intrinsic characteristics of the biomass. Phenolic compounds contribute to plant survival by protecting grains from both biotic and abiotic stress. Thus, it was expected that coffee beans would have a higher content of phenolic compounds, including flavonoids, compared to the layers surrounding the bean.^27^ Previous studies, such as that by Garret et al.,^78^ have already reported that beans attacked by coffee borers show a higher accumulation of flavonoids, functioning as a defense response by the plant. One hypothesis for the higher flavonoid content, given that the phenolic content remained the same, is that defective beans may have accumulated flavonoids as a defense mechanism, while other phenolics (such as phenolic acids) remained unchanged.

About antioxidant activity, in raw coffee beans, literature indicates a range from 5.03 to 253.96 mg TE·g^–1^ for DPPH inhibition^73,75,76^ and from 69.95 to 974.20 μmol TE·g^–1^ for FRAP.^73,74^ The values obtained in this study, described in Table S5, are within the above-mentioned arrays, indicating that the antioxidant compounds remain in the coproducts studied. This suggests that the extraction and/or processing did not result in significant losses of antioxidants. This preservation is of great importance, as it confirms the potential of those coffee coproducts as a valuable source of substances beneficial to health.

Moreover, a comparative analysis of the bioactive compounds and antioxidant activities in healthy and defective coffee beans residues reveals significant correlations between these parameters. The increase in total flavonoids content was accompanied by a higher FRAP value in the defective beans sample (*p* < 0.05), suggesting a positive correlation between flavonoid concentration and the ferric reducing antioxidant power. This reinforces the role of flavonoids as potent electron donors, capable of reducing ferric (Fe^3+^) to ferrous (Fe^2+^) ions, thereby contributing effectively to the antioxidant capacity. In contrast, no significant difference was observed in DPPH scavenging activity of both samples (5.06 vs 5.03 mg TE·g^–1^; *p* > 0.05), indicating that the radical scavenging mechanisms may be less sensitive to variations in specific classes of phenolic compounds.

Despite those slight differences, both types of residual coproducts retain comparable antioxidant potential, highlighting their possible valorization in functional or nutraceutical applications.

In summary, the results obtained reinforce the viability of the biorefinery concept applied to defective coffee beans, demonstrating that even though they are considered to have low commercial value, these residues retain compounds of nutritional and functional interest that can be directed to different production chains. The waste studied here showed potential for application in different sectors, especially due to the presence of fibers, proteins, amino acids, and bioactive compounds. In the food sector, they can be exploited as functional flours or antioxidant ingredients, provided that the caffeine content is controlled, and the formation of acrylamide in thermal processes is mitigated. For animal feed, they stand out as sources of fiber and fermentable sugars, observing regulatory limits for caffeine. In nutraceutical and pharmaceutical applications, the presence of antioxidants and essential amino acids suggests potential for supplements and functional formulations, subject to proof of toxicological safety and specific regulatory frameworks. Thus, the full use of this material contributes to waste reduction, adds economic value, and promotes practices aligned with the circular economy, consolidating the role of coffee as a model for the development of large-scale biorefineries.

## Conclusions

The biorefinery concept can be effectively applied to coffee beans, especially defective ones, as a strategy for their valorization. The coffee oil extraction process and subsequent extraction of sugars from the cake proved to be successful, highlighting the viability of using these compounds.

The central focus of this work was the analysis of the extruded, defatted, and enzymatically hydrolyzed solid residue, revealing a remarkable potential for the extraction of proteins, fibers, and bioactive compounds. It was found that the content of these compounds remained practically unchanged compared to that of the bean not treated, which indicates that their functional properties were preserved during the extraction processes, except for a potential loss of amino acids from the original matrix.

These results are promising as they suggest that the waste generated during coffee production can be effectively integrated into the circular economy, generating yet another possibility for the use of defective grains to increase their value. By utilizing this waste to obtain compounds with high nutritional and functional value, this study contributes not only to the valorization of defective coffee beans but also to the promotion of sustainability in the coffee production chain. This approach could pave the way for new opportunities in the food and biomaterials industry, in line with the principles of the circular economy and the efficient use of natural resources.

## Supplementary Material



## Notes

Table S1 referring to data on monosaccharides found in healthy and defective raw coffee beans through acid hydrolysis; Table S2 refers to monosaccharides found in healthy and defective raw coffee beans through enzymatic hydrolysis for 72 h; Table S3 refers to the chemical composition of the samples (residual coproduct after hydrolysis of healthy and defective beans); Table S4 referring to the amino acid profile of the sample (residual coproduct of healthy and defective beans after hydrolysis); and Table S5 referring to the bioactive compounds and antioxidant activity of the sample (residual coproducts of healthy and defective beans after hydrolysis) (PDF)
