# Supplementary material for: Analysis of Waste Material from Enzymatic Hydrolysis of Extruded Arabica Coffee Beans from a Bioeconomy Perspective
Source: ACS Omega. 2025 Nov 11;10(46):55832–41. doi: 10.1021/acsomega.5c07362 (PMC12658715; doi:10.1021/acsomega.5c07362)
Supplement: Supplementary file 1 [file ao5c07362_si_001.pdf]

Analysis of waste material from enzymatic hydrolysis of extruded Arabica coffee beans from a bioeconomy perspective

*Raquel C. Ribeiro [a], Rita C. Alves [b], Liliana E. Santo [b], Anabela S. G. Costa [b], Susana Machado [b], M. Beatriz P. P. Oliveira [b], Ricardo S. S. Teixeira [c], Claudia M. Rezende [a]**

[a] Universidade Federal do Rio de Janeiro, Instituto de Química. Rua Athos da Silveira Ramos, 149, 6° andar, 626A - Cidade Universitária, Rio de Janeiro - RJ, Brasil, 21941-909.

[b] Universidade do Porto, Faculdade de Farmácia. Rua Jorge Viterbo Ferreira, 228, Porto - Porto, Portugal, 4050-313.

[c] Universidade Federal do Rio de Janeiro, Instituto de Química. Rua Moniz de Aragão, 1057 – Cidade Universitária, Rio de Janeiro - RJ, Brasil, 21941-914.

SUPORTING INFORMATION

Table S1. Monosaccharides found in healthy and defective raw coffee beans through acid hydrolysis

Sample	Monosaccharide composition (%)			
	Mannose+Fructose*	Galactose	Glucose	Arabinose
Healthy	19.69 ± 4.06 [a]	13.53 ± 0.70 [a]	6.56 ± 0.21 [a]	1.91 ± 0.38 [a]
Defective	18.50 ± 2.07 [a]	12.59 ± 0.76 [a]	5.52 ± 0.53 [a]	1.68 ± 0.13 [a]

Xylose was not found in the samples.

*Mannose and fructose content was described together due to limitations in the analytical procedure.

Values are expressed as mean ± standard deviation (n=3). Within each column, significant differences determined using the Student's t test for independent samples (p<0.05) are represented by different superscript letters.

Table S2. Monosaccharides found in healthy and defective raw coffee beans through enzymatic hydrolysis for 72 h

Sample	Monosaccharide composition (%)		
	Mannose+Fructose*	Galactose	Glucose
Healthy	11.67 ± 0.20 [a]	0.26 ± 0.02 [a]	5.21 ± 0.14 [a]
Defective	11.69 ± 0.09 [a]	0.26 ± 0.02 [a]	4.76 ± 0.07 [a]

Xylose and Arabinose was not found in the samples.

*Mannose and fructose content was described together due to limitations in the analytical procedure.

Values are expressed as mean ± standard deviation (n=3). Within each column, significant differences determined using the Student's t test for independent samples ($p<0.05$) are represented by different superscript letters.

Table S3. Chemical composition in the samples (residual co-product after hydrolysis) expressed in dry weight (moisture content of healthy and defective beans of 63.16 ± 0.08 $g.100^{-1}$ and 54.10 ± 0.15 $g.100^{-1}$, respectively).

Compound ($g.100g^{-1}$)	Residual co-product	
	Healthy	Defectives
Ash	7.49 ± 0.42 ^{a}	6.03 ± 0.16 ^{b}
Fat	1.76 ± 0.47 ^{a}	0.86 ± 0.05 ^{b}
Crude protein	15.10 ± 0.12 ^{a}	14.99 ± 0.12 ^{a}
Nitrogen content	2.88 ± 0.02 ^{a}	2.86 ± 0.02 ^{a}
Protein nitrogen	2.42 ± 0.04 ^{a}	2.22 ± 0.03 ^{b}
Non-protein nitrogen	0.45 ± 0.02 ^{b}	0.64 ± 0.03 ^{a}
Fiber	56.48 ± 0.16 ^{a}	50.30 ± 0.24 ^{b}
Insoluble fiber	52.52 ± 0.36 ^{a}	47.56 ± 0.39 ^{b}
Soluble fiber	3.96 ± 0.37 ^{a}	2.74 ± 0.15 ^{b}
*Remaining carbohydrates**	16.27 ± 0.82 ^{b}	24.96 ± 0.14 ^{a}
Caffeine	0.94 ± 0.01 ^{a}	1.02 ± 0.05 ^{a}
Chlorogenic acid		
3-CQA	0.37 ± 0.01 ^{b}	0.45 ± 0.02 ^{a}
4-CQA	0.50 ± 0.01 ^{b}	0.70 ± 0.05 ^{a}
5-CQA	1.78 ± 0.04 ^{b}	2.44 ± 0.13 ^{a}

* Calculated using the following formula: 100% - (%ash + %raw protein + %fat+ % total fiber); 3-CQA: 3-O-caffeoylquinic acid; 4-CQA: 4-O-caffeoylquinic acid; 5-CQA: 5-O-caffeoylquinic acid.

Values are expressed as mean ± standard deviation (n=3). Within each line, significant differences determined using the Student's t test for independent samples ($p<0.05$) are represented by different superscript letters.

Table S4. Amino acid profile in the sample (residual co-product from healthy and defective grains after hydrolysis) in dry weight.

Compound	Residual co-product from healthy grains		Residual co-product from defective grains	
	Total amino acids (mg.g^{-1})	Free amino acids (µg.g^{-1})	Total amino acids (mg.g^{-1})	Free amino acids (µg.g^{-1})
Aspartic acid	13.44 ± 0.70 a	29.59 ± 4.33 A	14.44 ± 0.36 a	15.80 ± 1.33 B
Glutamic acid	24.45 ± 1.25 a	365.15 ± 3.79 B	25.55 ± 0.65 a	945.51 ± 12.91 A
Asparagine	n.d.	3.74 ± 0.54 B	n.d.	113.99 ± 1.89 A
Serine	6.95 ± 0.36 a	4.93 ± 1.27	7.10 ± 0.14 a	6.56 ± 0.64
Glutamine	n.d.	20.78 ± 0.88 B	n.d.	35.87 ± 2.43 A
*Histidine	2.42 ± 0.20 a	52.77 ± 1.41 A	1.67 ± 0.09 b	11.89 ± 2.28 B
Glycine	9.06 ± 0.55 a	51.23 ± 2.33 B	8.81 ± 0.21 a	74.77 ± 2.86 A
*Threonine	5.28 ± 0.24 b	67.79 ± 1.14 B	6.04 ± 0.15 a	116.53 ± 1.33 A
*Arginine	9.24 ± 0.47 a	286.07 ± 8.17 A	7.78 ± 0.34 b	22.91 ± 2.58 B
Alanine	6.86 ± 0.36 b	165.69 ± 11.79 B	8.34 ± 0.22 a	318.89 ± 5.06 A
Tyrosine	3.69 ± 0.22 a	82.35 ± 2.62 B	3.59 ± 0.16 a	178.22 ± 6.10 A
*Valine	7.43 ± 0.42 a	9.68 ± 1.35 B	8.21 ± 0.28 a	249.39 ± 2.99 A
*Methionine	0.67 ± 0.07 b	9.59 ± 1.18 B	0.82 ± 0.05 a	31.95 ± 2.59 A
*Tryptophan#	1.29 ± 0.00 a	14.69 ± 1.38 B	1.13 ± 0.06 b	42.29 ± 1.95 A
*Phenylalanine	10.37 ± 0.60 a	188.52 ± 1.94 B	11.12 ± 0.24 a	320.77 ± 7.84 A
*Isoleucine	5.51 ± 0.28 b	51.06 ± 0.88 B	6.99 ± 0.24 a	120.54 ± 3.81 A
*Leucine	16.90 ± 0.83 b	227.91 ± 2.24 B	18.76 ± 0.49 a	528.55 ± 10.37 A
*Lysine	8.62 ± 0.44 a	316.68 ± 18.50	8.41 ± 0.16 a	281.90 ± 5.99

Hydroxyproline	0.97 ± 0.03 a	20.91 ± 0.38 B	1.03 ± 0.02 a	24.73 ± 0.74 A
Proline	8.30 ± 0.36 b	262.95 ± 5.55 A	9.13 ± 0.14 a	223.10 ± 3.38 B
∑ Total amino acids (TAA)	141.47 ± 7.22	2232.08 ± 35.47 B	148.92 ± 3.30	3664.20 ± 49.14 A
*∑ Essential amino acids	58.50 ± 2.95	938.70 ± 19.41 B	63.14 ± 1.30	1703.82 ± 19.06 A

n.d. not detected (conversion of asparagine and glutamine to aspartic acid and glutamic acid during acid hydrolysis, respectively), # determined by basic hydrolysis. Within each line, different lowercase letters (a and b) represent significant differences ($p<0.05$) between the total amino acid content of healthy and defective samples, determined using the Student's t test for independent samples ($p<0.05$). In turn, different uppercase letters denote significant differences ($p<0.05$) between the free amino acid content of healthy and defective samples.

Table S5. Bioactive compounds and antioxidant activity of the sample (residual co-products from healthy and defective grains after hydrolysis) in dry weight, expressed as mean ± standard deviation.

Residual co-product	Bioactive compounds		Antioxidant activity	
	Total phenolics (*mg CAE.g^{-1}*)	Total flavonoids (*mg CE.g^{-1}*)	FRAP (*µmol FSE.g^{-1}*)	DPPH$^{\bullet}$-SA (*mg TE.g^{-1}*)
Healthy	44.01 ± 2.71 ^{a}	9.60 ± 1.54 ^{b}	522.44 ± 8.95 ^{a}	5.03 ± 1.37 ^{a}
Defective	41.19 ± 4.93 ^{a}	15.14 ± 1.70 ^{a}	545.27 ± 12.78 ^{a}	5.06 ± 0.57 ^{a}

Values are expressed as mean ± standard deviation (n=9). Within each column, significant differences determined using the Student's t test for independent samples ($p<0.05$) are represented by different letters. FRAP: ferric reducing antioxidant power; DPPH●-SA: DPPH radical scavenging ability; CAE: chlorogenic acid equivalents; CE: catechin equivalents; FSE: ferrous sulphate equivalents; TE: trolox equivalents.
